# Oral colonization of *Acinetobacter baumannii* in intensive care units: Risk factors, incidence, molecular epidemiology, association with the occur of pneumonia and sepsis, and infection control measures

**DOI:** 10.22038/IJBMS.2022.59713.13243

**Published:** 2022-02

**Authors:** Yucel Duman, Yasemin Ersoy, Elif Seren Tanriverdi, Barıs Otlu, Sibel Altunisik Toplu, Harika Gözde Gözükara Bağ, Mehmet Sait Tekerekoglu, Nazire Bulam, Elif Kaplan Canturk, Nalan Parmaksiz

**Affiliations:** 1 Medical Microbiology Department. Inonu University Medical Faculty, Malatya, Turkey; 2 Infection Disease Department. Inonu University Medical Faculty, Malatya, Turkey; 3 Biostatistics and Medical Informatics Department. Inonu University Medical Faculty, Malatya, Turkey; 4 Infection Control Committee, Turgut Ozal Medical Center, Malatya, Turkey

**Keywords:** Acinetobacter baumannii, Infection control measures, Intensive care units, mcr-2, Oral colonization

## Abstract

**Objective(s):**

Oral colonization of *Acinetobacter baumannii *can lead to infections such as pneumonia and sepsis. We aimed to evaluate oral colonization of hospitalized patients in ICUs and to examine risk factors for oral colonization, molecular epidemiology, and incidence of pneumonia and sepsis.

**Materials and Methods:**

The study began in February 2021. Oral cultures were taken. The microorganisms were identified by a Maldi-tof MS mass spectrometry device. Colistin resistance genes were investigated by polymerase chain reaction. Clonal relationships were determined by pulsed-field gel electrophoresis.

**Results:**

*A. baumannii *was found in 21 of 96 patients’ oral cultures. Pneumonia and sepsis due to *A. baumannii *were detected in 14 and 5 patients, respectively. The mean growth time of *A. baumannii* from oral cultures was 11.8 days, and the meantime for the occurrence of pneumonia after oral growth was 5.2 days. We determined a plasmid mediated mcr-2 colistin resistance gene in a colistin susceptible *A. baumannii *strain. It is the first report of the plasmid mediated mcr-2 colistin resistance gene in our country. In total, fourteen different *A. baumannii *genotypes were determined in PFGE. It was determined that the effects of antibiotic use, oral motor dysfunction, mechanical ventilation, intubation, orogastric tube use, and total parenteral nutrition intake on oral colonization were statistically significant.

**Conclusion:**

Oral colonization of *A. baumannii *is a significant concern in ICUs. We believe that it is important to take oral cultures and follow the risk factors and take infection control measures to prevent oral colonization of resistant isolates in ICUs.

## Introduction

Infections in intensive care units (ICUs) cause high mortality and morbidity due to antimicrobial resistance of the causative microorganisms. These infections account for about half of all healthcare-associated infections (HCAIs) (1). *Acinetobacter baumannii* is one of the most common causative microorganisms in ICUs (2-3). It can cause a wide variety of serious HCAIs such as sepsis, pneumonia, meningitis, necrotizing fasciitis in immunocompromised, ventilator-dependent patients (3-5). It is among the red list group of ESKAPE pathogens declared as critical priority pathogens by the World Health Organization (4). Bacteria in this group are important because they have multi-drug resistance (MDR) and cause epidemics (5, 6).

Colistin is used as a last resort in the treatment of infections caused by multi-drug resistant *A. baumannii*. The emergence of plasmid-mediated colistin resistance is a serious situation in terms of increasing the horizontal spread of colistin resistance among bacteria, and these genes are a potential issue for public health (7). Correct identification of mcr-encoding isolates is critical to limiting the spread because mcr-mediated colistin resistance is transferable between bacterial strains, species, and genera (8). 

Many factors lead to colonization of *A. baumannii*, especially in ICUs. HCAIs depend on risk factors such as prolonged ICU stay, mechanical ventilation, intubation, and colonization (5, 9). Oral colonization is one of the most important risk factors for pneumonia and sepsis in these patients. It has been reported that *A. baumannii* is responsible for 3–20% of HCAIs and the mortality rate of *A. baumannii*-related infections in ICUs is between 50–60% (1, 10). *A. baumannii* is resistant to many stress factors, such as dryness and disinfectants (10). These properties, combined with its ability to form biofilms on surfaces, increase the possibility of the microorganism remaining in medical equipment and hospital settings, making it particularly dangerous for immunocompromised ICU patients (10,11). The CDC stated that contact transmission direct from body surface to body surface or indirect transmission via contaminated inanimate objects is one of the main routes of microorganism transmission. *A. baumannii* can be easily colonize the inanimate surfaces and survive for several months (10). Bacterial transmissions mainly come from contact with contaminated surfaces, but they can also be spread from person to person in hospitals. Oral colonization of *A. baumannii *in ICU patients may lead to its transmission to the lower respiratory tract, causing pneumonia and other respiratory tract infections. Researching, identifying, and evaluating the risk factors that cause HCAIs are important themes that will guide studies and education programs on infection control. In addition, evaluation of the risk factors of oral *A. baumannii* colonization will help reduce the mortality of patients hospitalized in ICUs and increase the quality of infection control measures. 

In this study, we aimed to evaluate oral colonization of hospitalized patients in ICUs and to examine risk factors for oral colonization, the incidence and molecular epidemiology of *A. baumannii* and its relationship with the occurrence of pneumonia and sepsis, and infection control measures.

## Materials and Methods

***Setting and definition***

The study was conducted in a 1300-bed capacity university hospital in Eastern Turkey. There are 16 ICUs and 300 ICU beds in the hospital. The study was started at the beginning of February 2021. An oral colonization analysis was begun to determine the risk factors and definition of the cases. Retrospective and prospective analyses were undertaken. A table was created to determine the risk factors. Data on patients, including age, gender, underlying diseases, oral motor dysfunction, antibiotic use, mechanical ventilation, invasive procedures, ICU hospitalization days, and other risk factors were collected from electronic records, patients’ files, and medical personnel. 

Oral cultures were taken from all inpatients in ICUs on the first day of the study to determine the existence of oral colonization. Oral colonization determined inpatients on the first day were excluded from the study (as the time of colonization could not be determined). Newly hospitalized patients in the ICUs and inpatients whose oral colonization was not determined were included in the study. Oral cultures were taken every day from newly hospitalized patients and inpatients whose oral colonization was not determined. Patients with pathogenic microorganism growth in their oral culture were followed for 30 days for the occurrence of pneumonia and sepsis.

***Bacterial strains: Source, identification, and susceptibility tests***

Oral cultures were taken with sterile swab sticks and inoculated on sheep blood agar and eosin methylene blue agar. *Streptococci* and coagulase-negative *staphylococci* were excluded from the growing samples, since they could be members of the oral flora, and pure passages of pathogenic microorganisms that could be infectious agents were taken and identified by conventional methods and a matrix-assisted laser desorption ionization time-of-flight mass spectrometry device (Maldi-tof MS) (BioMérieux, France). Antimicrobial susceptibility tests were performed with a Vitek-2 (Biomérieux, France) automated device. Minimal inhibitory concentrations (MICs) of colistin were studied with the standard broth microdilution (BMD) method. BMD was performed using 96-well broth microdilution panels according to the European Committee on Antimicrobial Susceptibility Testing (EUCAST) guide (12). The BMD MIC test range was determined as 32 μg/ml–0.06 μg/ml for colistin. Results were evaluated according to the EUCAST criteria.

**Detection of colistin resistance genes**

The presence of mobile colistin resistance genes was screened using in-house PCR. The presence of the mcr 1-5 gene regions responsible for plasmid-mediated ColR was determined using in-house multiplex PCR, as Rebelo *et al*. (13) suggested. The primer sets are as follows: mcr-1 (F) 5’-AGTCCGTTTGTTCTTGTGGC-3’ mcr-1 (R) 5’-AGATCCTTGGTCTCGGCTTG-3’, mcr-2 (F) 5’-CAAGTGTGTTGGTCGCAGTT-3’ and mcr-2 (R) 5’-TCTAGCCCGACAAGCATACC-3’, mcr-3 (F) 5’-AAATAAAAATTGTTCCGCTTATG-3’ and mcr-3 (R) 5’-AATGGAGATCCCCGTTTTT-3’, mcr-4 (F) 5’-TCACTTTCATCACTGCGTTG-3’ and mcr-4 (R) 5’-TTGGTCCATGACTACCAATG-3’, mcr-5 (F) 5’-ATGCGGTTGTCTGCATTTATC-3’ and mcr-5 (R) 5’-TCATTGTGGTTGTCCTTTTCTG-3’. DNA extraction of isolates was performed with a column-based DNA isolation kit (DNA mini kit, Qiagen, Germany). The PCR run was performed using the GeneAmp PCR System 9700 device (Applied Biosystems). Amplicons were photographed with ultraviolet illumination after electrophoresis.

***Molecular typing***

The clonal relationships of *A. baumannii *isolates were determined by pulsed-field gel electrophoresis (PFGE). The APA-I enzyme was used as a cutting enzyme. Band profiles were analyzed using the GelCompar II software system (version 6.5; AppliedMaths, Sint-Martens-Latem, Belgium). The dice correlation coefficient was used to calculate similarity for band analysis, and the UPGMA (“Unweighted Pairwise Grouping Mathematical Avenging”) method of grouping unweighted pairs with mathematical mean was used for cluster analysis. Considering the similarity coefficients of the isolates, the strains showing more than 90% similarity with each other were accepted in the same or different clones. Strains with similarity rates below 90% were evaluated as different or separate from the others (14).

***Statistical methods***

The distribution of the qualitative data was represented as a count and percentage. Comparisons according to these variables were performed by continuity-corrected chi-square (two-sided) or Fisher’s exact test (one-sided), where appropriate.

## Results

A total 96 of patients were included in the study. 50 (52.1%) of the patients were newly hospitalized, and 46 (47.9%) were inpatients whose oral colonization was not determined at the beginning of the study in the ICUs. 54 (56.2%) of the patients included in the study were male and 42 (43.8%) were female. The mean age was 63.59 (20–90) (sd: 16,224). Oral care was applied to all patients with sodium bicarbonate and chlorhexidine 4–6 times a day. 

*A. baumannii* was found in 21 of 96 (21,9%) patients by oral culture. Also, other pathogenic microorganisms were found in 16 of 96 (16,6%) patients’ oral cultures. Pathogenic microorganism growth could not be determined in the oral cultures of 59 (61,5%) patients. Only *A. baumannii* was isolated in the oral cultures of 18 patients. In 3 patients, *Klebsiella pneumoniae* was isolated together with *A. baumannii*. Pathogenic microorganisms isolated from oral culture are shown in Table 1. Pneumonia due to *A. baumannii* was found in 14 of the patients. *A. baumannii* sepsis was detected in five patients. Pneumonia incidence rates and causative microorganisms are shown in Table 2. The mean growth time from oral cultures for all colonized microorganisms was 10.2 days, and the mean growth time from oral cultures for *A. baumannii* was 11.8 days after the patients were admitted to the intensive care unit. The mean time for the occurrence of pneumonia after oral growth was 5.2 days.

When the risk factors were examined, it was determined that the effects of antibiotic use, oral motor dysfunction, mechanical ventilation, intubation, orogastric tube use, and total parenteral nutrition intake on oral colonization were statistically significant. The effects of risk factors on oral colonization are shown in Table 3.

According to EUCAST, all *A. baumannii* isolates were susceptible to colistin (MICs 0.125 to 2 μg/ml) but resistant to aminoglycosides, quinolones, cephalosporins, carbapenems. Colistin MIC_50_ and MIC_90_ were determined as 0.5 μg/ml and 2 μg/ml, respectively. We determined a plasmid mediated mcr-2 colistin resistance gene in a colistin susceptible* A. baumannii* strain. This is the first report of the plasmid mediated mcr-2 colistin resistance gene in Turkey. Also, we verified it by cycle sequencing.

**Figure 1:**
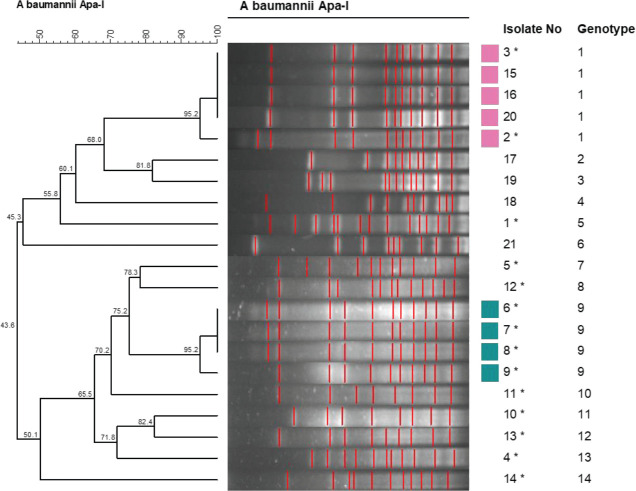
Dendrogram of *Acinetobacter baumannii* isolated from oral cultures

Fourteen different *A. baumannii* genotypes were determined in PFGE performed to determine the clonal relationship of the patient’s oral cultures. The PFGE dendrogram is shown in Figure 1. Genotype 1 was detected in 5 patients, and genotype 9 was detected in 4 patients. Different genotypes were determined in other patients and there was no clonal relationship between them. When the relationship of risk factors for colonization of the same genotypes between these patients was examined; it was determined that they were hospitalized in the same ICU, mechanical ventilation was applied, and they were intubated. This shows us that hand hygiene and environmental cleanliness are very important because *A. baumannii* stays on surfaces for a long time and causes outbreaks. 

***Infection control interventions***

As an infection control measure, retraining was organized for the personnel. In training, hand hygiene rules, environmental and device cleaning, importance of *A. baumannii*, and rules to be followed in the epidemic were explained. When we determined the mcr-2 colistin resistance gene, infection control measures such as tight contact prevention, good clean-up of the hospital environment and medical devices, and staff training in ICUs were taken to prevent its quick spread . Hand hygiene and disinfection training programs were created to educate staff. During the staff work, observations and feedback were taken by the infection control nurse.

**Table 1:** Distribution of microorganisms isolated from oral cultures

	Frequency	Percent %
*Acinetobacter baumanii*	21	56.8
*Klebsiella pneumoniae*	10	27
*Staphylococcus aureus*	1	2.7
*Pseudomonas aeruginosa*	2	5.4
*Stenotrophomonas maltophilia*	3	8.1
Total	37	100

**Table 2:** Examination of pneumonia with oral growth and causative microorganisms

		**Pneumonia**		
		no n (%)	yes n (%)	*P*
Oral growth of microorganisms	no	58 (98.3)	1 (1.7)	<0.001
	yes	19 (51.4)	18 (48.6)	
Causative microorganisms	*A. baumannii*	7 (33.3)	14 (66.7)	0.029
	Others	12 (75)	4 (25)	

**Table 3:** Examination of the correlation between the risk factors and growth of microorganisms in oral cultures

			**Oral growth** **of microorganisms**		
			no n (%)	yes n (%)	*P*
**Risk factors**					
Comorbidities		no	25 (58.1)	18 (41.9)	0.696
		yes	34 (64.2)	19 (35.8)	
Diabetes		no	42 (65.6)	22 (34.4)	0.236
		yes	17 (53.1)	15 (46.9)	
Dental prosthesis		no	41 (61.2)	26 (38.8)	1.000
		yes	18 (62.1)	11 (37.9)	
Oral motor dysfunction		no	35 (79.5)	9 (20.5)	0.002
		yes	24 (46.2)	28 (53.89	
Mechanic ventilation		no	38 (80.9)	9 (19.1)	<0.001
		yes	21 (42.9)	28 (57.1)	
Antibiotic use		no	18 (94.7)	1 (5.3)	0.001
		yes	41 (53.2)	36 (46.8)	
Intubation		no	37 (80.4)	9 (19.6)	0.001
		yes	22 (44)	28 (56)	
TPN		no	35 (76.1)	11 (23.9)	0.009
		yes	24 (48)	26 (52)	
NG use		no	41 (68.3)	19 (31.7)	0.116
		yes	18 (50)	18 (50)	
OG tube use		no	57 (64.8)	31 (35.2)	0.035*
		yes	2 (25)	6 (75)	

*Fisher’s exact test one-sided *P*-value, TPN: Total Parenteral Nutrition, NG: Nasogastric, OG: Orogastric

* Isolates of patients with pneumonia

## Discussion

Oral colonization of inpatients in ICUs by *A. baumannii* and other multidrug-resistant pathogenic bacteria is a significant concern as they increase the treatment failure. Furthermore, colonization of the oral cavity plays an important role in the spread of these microorganisms to other inpatients and the hospital environment (2,3,15). Twenty-one *A. baumannii* isolates were identified as oral colonization of patients. All strains were susceptible to colistin but resistant to carbapenems, aminoglycosides, cephalosporins, and quinolones. 

Gram-negative microorganisms colonize the oral cavity of less than 10% of healthy individuals. In ICU inpatients, this rate rises to 75%. Johnstone *et al*. (16) reported that 41% of oral culture samples taken from children 24 hr after hospitalization in the ICU were colonized with pathogenic bacteria. In our study, we determined the oral colonization of pathogenic microorganisms in 37 (38.5%) of 96 patients. Among the colonized microorganisms, *A. baumannii* was found to be the most common, with 56.8% (Table 1). The mean growth time for all colonized microorganisms (10.2 days) and *A. baumannii* (11.8 days) was more than ten days. This shows us that prolonged ICU stays are an important risk factor for oral colonization. 

The presence of microorganisms such as *A. baumannii*, *Paeruginosa aeruginosa*, *K. pneumoniae*, *Staphylococcus aureus* in the oral cavity poses a potential risk for pulmonary and bloodstream infections. It is clear that the oral cavity is one of the main foci for the spread of potentially pathogenic microorganisms to other body parts as a result of oral colonization. The lung is the most affected organ due to mechanical ventilation, which is one of the most important risk factors in this spread (17-19). According to studies the most common bacteria causing pneumonia in ICUs are *P. aeruginosa* (30.1%), *S. aureus* (19.6%), *Acinetobacter spp.* (13.0%), *Klebsiella spp.* (9.5%), and *Enterobacter spp.* (8.4%) (19,20). Azim *et al*. (21) reported in their study that 73% of the microorganisms colonizing patients in ICUs were *P. aeruginosa* or *Acinetobacter*. Mechanical ventilation, antibiotic use, and hospital stay longer than 48-hours were also risk factors. On the other hand, a study (22) reported that *A. baumannii* was responsible for 29.4% of VAPs. Available data in the literature show that mortality after pneumonia due to *A. baumannii* varies between 26% and 68% (22-24). In our study, pneumonia was found in 19 of 96 patients. Oral colonization was present in 18 of the patients with pneumonia. We determined that 66.7% of *A. baumannii* strains isolated from oral cultures caused pneumonia, and the meantime to the occurrence of pneumonia after oral growth was less than a week (5.2 days). Among other pathogens, we found that two strains of *K. pneumoniae*, one *S. aureus* and one *P. aeruginosa* cause pneumonia. We determined a statistical association between oral colonization and pneumonia (*P*<0.001) (Table 2). When the cause of pneumonia due to *A. baumannii* was compared with other bacterial agents, it was determined that the occurrence of pneumonia due to *A. baumannii* was statistically significant (*P*=0.029) (Table 2). Sepsis due to *A. baumannii* was found in five patients and due to *K. pneumoniae* in three patients.

*A. baumannii* causes significant treatment problems*. A. baumannii* is now recognized as one of the main factors in the antimicrobial resistance crisis. It is also the most common opportunistic pathogen in ICUs. The Central Asian and Eastern European Antimicrobial Resistance Surveillance Network (CAESAR) reported that the rates of *Acinetobacter* isolated from invasive specimens in ICUs were 23% in 2017 and 21% in 2018 in the world. In addition, it is reported that the rates of *Acinetobacter* with MDR (quinolones, aminoglycosides, and carbapenems) worldwide were 57% in 2017 and 62% in 2018. In Turkey, the rate of *Acinetobacter* with MDR was 76% in 2017 and 78% in 2018. In the 2017 CAESAR report, carbapenem resistance was reported as 92% in *A. baumannii* isolates in our country, and 91% in the 2018 report (25,26). In our study, all of the isolates were resistant to carbapenem group antimicrobials, aminoglycosides, quinolones, and cephalosporins. The emergence of mobile colistin resistance genes and the formation and spread of colistin resistance has become an important health problem. After the declaration of mcr-1 in China, reports on mcr genes were published from various parts of the world. In our study, we determined the mcr-2 gene in one *A. baumannii* isolate which is susceptible to colistin. This is the first report in our country. When we examined this isolate, we determined that it caused pneumonia and sepsis in the patient it had orally colonized. After determination of mcr-2 positive *A. baumannii*, we first performed tight contact prevention and staff training as infection control measures to prevent the quick spread of the mcr-2 gene. Also, hand hygiene and disinfection training programs were created to educate staff. During the staff work, observations and feedback were taken by the infection control nurse.

Among the 21 *A. baumannii* strains, no significant outbreak isolates were identified by PFGE (Figure 1). But it was determined that oral colonization of the patients with pneumonia and the strains thought to be the cause of pneumonia isolated from sputum and tracheal aspirate cultures were the same strains as PFGE. When we evaluated it statistically, we found that there was a significant relationship between pneumonia and oral colonization due to *A. baumannii*. When the relationship of risk factors for colonization of the same genotypes between these patients was examined, it was determined that they were hospitalized in the same ICU, mechanical ventilation was applied, and they were intubated. This shows us that hand hygiene and environmental cleanliness are very important for *A. baumannii* to stay on surfaces for a long time and cause outbreaks. As an infection control measure, retraining was organized for the staff. In training, hand hygiene rules, correct hand hygiene, environmental and device cleaning, the importance of *A. baumannii*, and the rules to be followed in the epidemic were explained.

Oropharyngeal colonization of “non-oral” pathogenic microorganisms in ICUs depends on many risk factors such as underlying diseases, mechanical ventilation, orogastric use, presence of TPN, intubation, and long-term antibiotic use. When we examined the risk factors for colonization, we determined that there is a statistically significant relationship between oral motor dysfunction, mechanical ventilation, intubation, TPN, OG, antibiotic use, and oral colonization (Table 3). In various studies conducted in our country and around the world, it is stated that oral colonization is an important factor for the development of pneumonia. It is mentioned that the use of antibiotics for oral colonization, mechanical ventilation, and length of hospital stay are the most important risk factors (3, 22, 23). 

We believe that infections due to *A. baumannii*, which have increased in the last decade, are easily colonized in the hospital environment and are difficult to eradicate after colonization. In our study, especially *A. baumannii* reveals that oral colonization increases in ICUs and is very important for the incidence of pneumonia and the related sepsis.

Oral hygiene is considered a traditional method of routine patient care for the prevention of HCAIs. Many studies show that routine oral hygiene reduces oropharyngeal colonization compared with non-invasive practices and can reduce the incidence of nosocomial infections in ICU inpatients. The Centers for Disease Control and Prevention (CDC) recommends oral care in the ICUs. In line with the recommendations of the infection control committee in our hospital, oral care is applied in our ICUs with chlorhexidine and bicarbonate 4–6 times a day. However, in our study, we detected *A. baumannii* oral colonization in 21 of our patients during the follow-up period. We detected pneumonia due to *A. baumannii* in 14 of these patients, and sepsis in 5 of them. This made us think that oral care practice was inadequate or unsuitable for *A. baumannii* colonization. In a study, Umezawa *et al*. (27) reported that *A. baumannii* colonized the tap water and thus caused oropharyngeal colonization during oral care and then an epidemic. In this context, procedures have been established for oral care practices for staff. In oral care, it is recommended to use sterile water instead of tap water. The use of alcohol-based hand antiseptics before and aftercare was provided. Awareness was created by explaining the importance of oral care, colonization of *A. baumannii*, and prevention of HCAIs through training.

## Conclusion

Oral colonization of* A. baumannii* and other pathogenic bacteria is a significant concern in ICUs, and the morbidity and mortality of infections caused by these bacteria are high. Aside from colonization, these bacteria have increased resistance to antimicrobials. In addition, we believe that it is important to take oral cultures and follow the risk factors of these isolates in ICUs and take necessary infection control measures to prevent the oral colonization of resistant isolates in hospitals.

## Authors’ Contributions

DY and EY Helped with study conception and design; DY, TMS, BN, KCE, PN, and TES Performed data analysis and prepared the manuscript draft; DY, OB, and GHG Critically revised the paper; OB, TMS, GHG, and ATS Supervised the research; DY, YE, TES, OB, ATS, GHG, TMS, BN, KCE, and PN Approved the final version to be published.

## Ethical Approval

This work was approved by Inonu University Scientific Research and Publication Ethics Board with decision number 2021/1696.

## Conflicts of Interest

The authors declare that there are no conflicts of interest.
